# Temperature-induced microstructural evolution and fractal characteristics of high-enthalpy Chumathang granite for enhanced geothermal energy

**DOI:** 10.1038/s41598-025-00683-2

**Published:** 2025-05-27

**Authors:** Mrityunjay Singh, Sachchida nand Pandey, Debanjan Chandra, Nishant Singh, Adarsh Tripathi, Sunil Kumar Yadav, Ingo Sass, Ajeet Kumar Srivastav, Sandip Kumar Saha

**Affiliations:** 1 Section 4.3 Geoenergy, GFZ Potsdam, 14473 Potsdam, Germany; 2 Department of Mechanical Engineering, Indian Institute of Technology Bombay, Mumbai, 400076 India; 3 Faculty of Civil Engineering and Geosciences, Technical University Delft, 5048 Delft, The Netherlands; 4 Department of Earth Sciences, Indian Institute of Technology Bombay, Mumbai, 400076 India; 5 Department of Earth Sciences, Indian Institute of Technology Roorkee, Roorkee, 247667 India; 6 Institute of Applied Geosciences, Geothermal Science and Technology, Technical University, Darmstadt, Germany; 7 Department of Metallurgical & Materials Engineering, Visvesvaraya National Institute of Technology, Nagpur, India

**Keywords:** Enhanced geothermal systems, Thermal cracking, Fractal dimensions, Mesopores, Surface area, Chumathang granite, Geothermal energy, Geochemistry, Energy access, Hydrogeology

## Abstract

Micro-structural attributes of Chumathang granite from Leh, India, were experimentally determined in the temperature range from 25 to 600 °C for enhanced geothermal systems (EGS). P-wave velocity, thermal crack generation, and pore attributes were analyzed using a combination of pulse ultrasonic velocity study, 3D X-ray tomography and low-pressure gas adsorption experiments, respectively. Results indicate that thermal crack development is driven by mineral composition and differential thermal expansion, with a significant increase in the thermal damage factor between 450 $^\circ{\rm C}$ and 600 $^\circ{\rm C}$, accompanied by visible cracks at 600 $^\circ{\rm C}$. Surface area and pore volume decreased up to 300 $^\circ{\rm C}$ due to mineral dissolution, then slightly increased up to 600 $^\circ{\rm C}$ due to microfracture formation. Pore size distribution showed a dominance of coarser mesopores, and fractal dimensions decreased with temperature, reflecting simpler pore geometries. These findings enhance the understanding of granite’s microstructural changes under thermal stress, informing the optimization of EGS heat extraction efficiency.

## Introduction

Enhanced geothermal system (EGS) is considered as one of the most promising sources of cleaner energy, that has gained importance in electricity generation and heating/cooling applications^1–3^. Among all the types of reservoir formations, the granitic reservoir is considered as an excellent medium for geothermal heat extraction, for having low water–rock interaction and slower reactive kinetics of the constituent minerals^4^. Some of the well-known enhanced geothermal systems (EGS) projects exist in granitic reservoir systems^5,6^. For clarity, the term granite is employed here in the practical engineering sense that covers quartz‑bearing granitoid rocks sensu lato. Quantitative modal analysis of thin sections from the Chumathang intrusive suite locates the studied specimens in the monzodiorite field of the IUGS QAP diagram^7,8^. This petrographic refinement does not alter the thermo‑elastic parameters that control EGS performance, because elastic modulus, thermal expansion coefficient, and fracture toughness vary only marginally across the diorite-monzodiorite-granite compositional range^9,10^. We therefore retain granite throughout the study for continuity with prevailing EGS literature while acknowledging its precise lithological classification here.

The physical property of granite in the temperature range of ~ 150–500 °C and hydrogeological conditions at the depth range of 3–5 km are of particular interest for enhanced and ultra-high-temperature geothermal systems^11,12^. Previous studies have found that fracture formation during hydraulic fracturing^13–15^, fracture propagation^16^, fracture permeability evolution^17,18 ^and heat extraction are controlled by a wide range of parameters, including initial reservoir temperature, in-situ stress, grain size, porosity, permeability and mineralogical compositions^19–25^. Fractured reservoirs in granitic settings have gained significant attention for their role in Enhanced Geothermal Systems (EGS), particularly due to fracture networks’ ability to enhance permeability by orders of magnitude compared to intact granite^26,27^. Recent studies demonstrate that cyclic- and pulse-pumping injection schemes reduce breakdown pressures while promoting distributed fracture networks through stress relaxation at crack tips, which improves fluid circulation efficiency^26^. Field- and laboratory-scale experiments in granitic rock reveal that fatigue hydraulic fracturing generates complex fracture geometries with branching patterns, critical for sustaining heat exchange in EGS reservoirs^26,28^. Advanced numerical modeling has progressed substantially, with discrete element fluid-solid coupling algorithms now capturing mesoscale mineral heterogeneity’s impact on hydraulic fracture propagation in crystalline granite^28^. Concurrently, enhanced *J*-integral formulations address limitations in classical fracture mechanics by improving accuracy in viscosity-dominated regimes, enabling better prediction of fracture front evolution across stress regimes^29^. These computational advances complement seismic attribute fusion techniques that map fractured-cave reservoirs in granitic buried hills using coherence cubes and single-frequency attribute bodies, essential for characterizing reservoir heterogeneity^27^. Laboratory experiments combined with mine-scale hydraulic stimulation tests provide empirical validation, showing that cyclic injection protocols modify magnitude-frequency distributions of acoustic emissions (AEs), correlating with safer seismic profiles and optimized permeability enhancement^26,28^. This integrated understanding of thermo-hydro-mechanical-chemical processes informs stimulation strategies to manage thermal drawdown and extend EGS operational lifetimes, particularly in high-temperature crystalline reservoirs^26,28,29^.

Granite is a low-permeable, heterogeneous and porous geomaterial composed of a wide range of minerals such as albite, quartz, calcite, dolomite, feldspar, plagioclase, siderite, and pyrite. The mineralogical heterogeneity and interconnectivity of minerals leads to complex pore structures, due to the vast range of pore sizes. In addition, geochemical reactions of minerals modify the pore structures and pore sizes that are difficult for measurements and correct numerical simulations. The reactions involving dissolution increase the volume of total pore space while reactions involving precipitation reduce the volume of total pore space. Proper understanding of the heterogeneous pore structure, pore-size distribution and the surface area of granite is critical, in assessing the fluid flow during injection and production operation of geothermal system. In the past, direct imaging methods such as field emission scanning electron microscopy (FE-SEM), atomic force microscopy (AFM), computed tomography scanning (CT)^30–33^ and other indirect methods (such as mercury intrusion capillary porosimetry, low-pressure gas adsorption (N_2_ or CO_2_), and helium pycnometry) have been used to describe pore morphology of geomaterials. The indirect methods provide quantitative assessments of pore size distribution (PSD), specific surface area, pore diameter and pore volume parameters^34–36^. While the influence of temperature on the thermo-physical behavior of granite has been studied extensively in the literature, their impact on surface area, pore diameter, pore-size distribution and fractal dimension during heating is not well understood. It has been well documented that granite experiences significant loss in physical–mechanical properties with an increase in formation temperature^37–43^. The thermal behavior of fractured granites have highlighted the influence of temperature‐induced microstructural evolution on reservoir performance in enhanced geothermal systems. The temperature rise induces thermal stress, causing thermal cracks, resulting in the decrease of seismic velocity, elastic moduli, compressive and tensile strength of the rock. These studies also indicate that the strength of the rocks increases upto around 200 °C, which is due to the expansion of the rock minerals that decreases the porosity and closes the microcracks inside the rock. Further heating could promote cracks inside the grains and at the grain boundaries, may lead to mineral phase transitions as well. Differential thermal expansion among constituent minerals initiates the formation of microcracks at temperatures above 450 °C, as evidenced by marked reductions in P‐wave velocity^44^. These microcracks progressively coalesce into complex fracture networks whose geometrical complexity can be quantified via fractal analysis; indeed, our experimental results indicate a decrease in the fractal dimension with increasing temperature suggesting a transition toward simpler pore geometries that can significantly influence fluid transport and heat exchange. Homand-Etienne and Troalen^45^ showed that the microcracks develop faster in the granite crystals between 500 °C and 600 °C due to $\alpha$ quartz to $\beta$ quartz transition at 573 °C. They also showed that further temperature rise resulted in excessive damage of rock (by crack formation) by $\beta$ quartz to $\alpha$ cristobalite phase change, which is assumed to occur at a temperature of 870 °C. Glover et al.^46^ used acoustic emission (AE) technique to monitor the thermal cracking during heating and recorded a strong peak of microcracking at the phase transition temperature for quartz (573 °C). David et al.^47^ showed that crack density of Peyratte granite increased from 0.2% to 4.4% during heating from a room temperature to 600 °C. Kumari et al.^48^studied the effects of ‘cooling rate’ in granitic rock formation and they pointed out the rapid cooling of rock causes more damage as compared to the slower cooling of rock, thereby proving the importance of cooling effect on geothermal systems^20–22,49–51^. The cooling effect is extremely important for geothermal systems. In geothermal heat extraction, injection of cold water induces thermal cracks around the injection-well since faster cooling occurs there. The thermal front movement leads to a complex stress distribution in reservoir rocks due to thermal gradients along the flow direction and different magnitudes of mineral thermal expansion coefficients. Thus, a prior analysis and understanding of thermo-physical behavior of rocks is extremely important for sustainable utilization of geothermal resources.

The heat extraction performance of geothermal reservoir is mainly controlled by the mineralogical heterogeneities together with the evaluation of reservoir physical and transport properties. First, we performed experiments to show the surface area variation with temperature. The surface area plays an important role during heat extraction and the surface roughness induces the flow nonlinearity. These experiments were performed under controlled condition by considering all standard protocols followed in the field of reservoir engineering. The study is novel in many aspects: (i) focus on temperature effects on physical property, (ii) variation of surface area, pore width and pore volume with temperature, (iii) complex nature of fracture formation, and (iv) surface roughness. The results of this study provide valuable information for geothermal system during heat extraction under varying temperature range and reservoir conditions. The approach/methodology can be used and apply to different geological systems (shale reservoir, oil and gas recovery etc.).

## Materials and methods

### Sampling and preparations

**Fig. 1:**
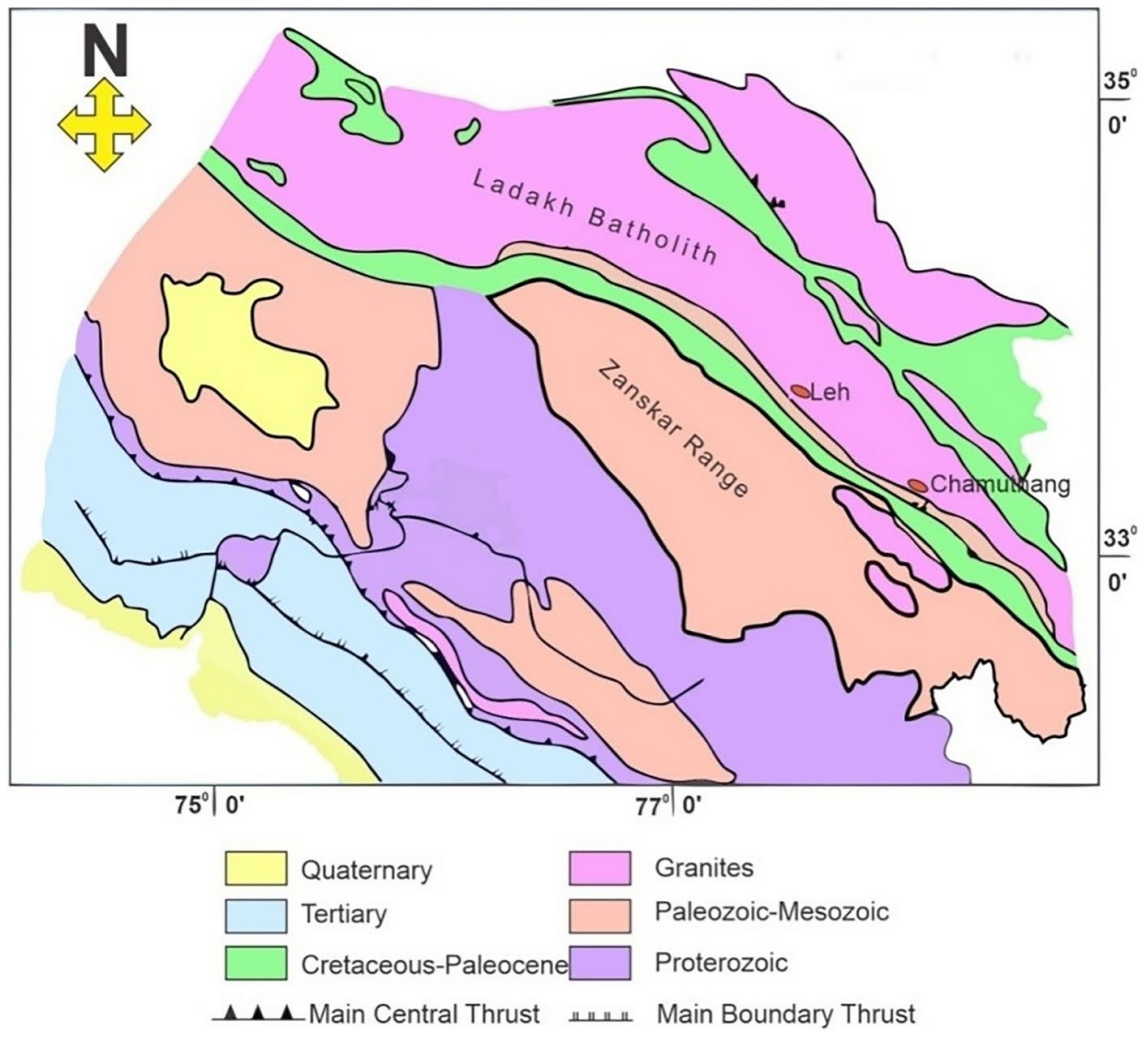
Regional stratigraphy of the field area with field area marked (after Craig et al.^52^).

In this experimental study**,** the granite samples were taken from high enthalpy Chumathang geo-thermal field, situated in Leh district, Ladakh, India (Fig. 1). Chumathang, located 138 km southeast of Leh on the northern bank of the Indus River, is known for its boiling springs, geysers, and hydrothermal deposits. The village covers an extent of 1 km^2^, with a near sub-surface and deeper sub-surface temperature of up to 130 °C and 260 °C, respectively^52^. At least, 73 hot springs and steaming-ground discharges can be found with travertine deposits indicating formal thermally active sites. The Indus Group sedimentary formation of Cenomanian to Miocene age in Chumathang geothermal site is intruded by Ladakh batholiths and Chumathang granite. The cumulative sodium-bicarbonate-chloride-type alkaline meteoric water discharge rate of Chamuthang is around 200 l/min, bearing an average temperature of 85 °C^53^. The granite samples were collected from the Chumathang village. The Chumathang granite represents an intrusive body within a post‐collisional granitic batholith that was emplaced during the later stages of Himalayan tectonic evolution. This granitic unit is intersected by a network of faults and fractures that are thought to control fluid pathways and enhance reservoir permeability in this high‐enthalpy setting. The sampling site was chosen from the central zone of the geothermal anomaly, where elevated heat flow, steep thermal gradients, and the presence of thermally induced microcracks have been documented^53^.

The objective of this study was to investigate the temperature-dependent physical characteristics of the selected Chumathang granite samples. The samples were divided in 5 different batches and each batch was heated at 4 different temperatures before further analysis. Cores, small blocks and powdered samples were prepared. Blocks of each of the batches were drilled to extract core samples (50 mm length and 25 mm diameter) as per the ISRM standard methods (Culshaw^54^) for the study of seismic wave velocity. For the 4D X-ray microscopy (4DXRM) investigations, cubic samples of 1 × 1 × 1 cm^3 ^were cut from the same 5 blocks of granite. 5-6 g samples from each batch were pulverized using agate mortar and further sieved using a 200 mesh to obtain a grain-size fraction of less than 70 μms. While, each set of cores, blocks and powdered samples from the same batch were heated at multiple temperatures with a constant heating rate of 5 °C/min for 24 h^47,55^, one set from the samples were kept at room temperature (Table 1). As different minerals in granites have varying thermal expansion coefficient, a faster/slower heating rate and lesser hold time would create a possibility of thermal stress, leading to fractures due to mismatch of thermal expansion^56,57^. Hence, the selected heating rate and the hold time of the temperature were sufficient for achieving the thermal equilibrium in the rock samples. Following the heating process, the oven was turned off after 24 h and the rocks were allowed to cool down slowly inside the oven (approx. 1 °C/min), negating any chance of fractures generated due to rapid cooling^57^. The seismic P-wave velocity of each core samples was measured before and after the heat treatment to neglect the impact of local heterogeneity. The powdered samples were used for the low-pressure gas adsorption (LPGA) and X-ray diffraction (XRD) studies.

**Table 1:**
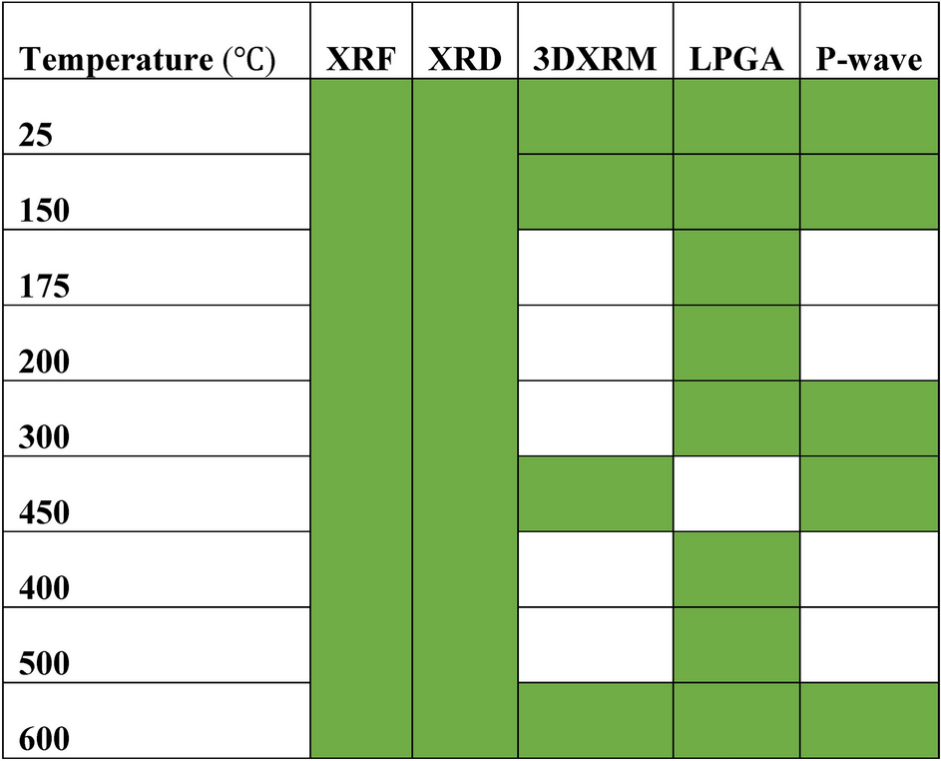
Treatment temperatures of granite samples and analysis performed for each sample (marked in green).

### X-ray fluorescence analysis

X-ray fluorescence (XRF) is one of the most important techniques for trace element analysis of granites. Fluorescence is generated based on the generation of characteristic X-ray from a sample irradiated by an X-ray beam. XRF is highly useful for its non-destructive properties, high detectability of elements with Z > 10 to 16 and low spectra interference. XRF analysis was conducted to investigate the elemental constituent in the rock samples. 8 g of powdered granite was mixed with 1.6 g wax and further pressed at 300 N/m^2^to form a pellet, that was subjected to analysis. Detailed analysis is explained by Helz and Taggart^58 ^and Oyedotun^59^.

### XRD analysis

The mineral composition of the granites was determined using a Malvern Panalytical X-ray diffractometer, equipped with a Cu anode and a Pixel 3D detector for high precision measurements. 2-4 mg of powdered granite sample was placed on the powder holder and flattened out into a disk shape for analysis and the scanning range was set between 5-70° 2θ angle for encompassing all mineral phases in granite with a scanning speed of 0.0136° per second. Detailed methodology is provided in^60^.

### Seismic wave velocity measurement

The P-wave velocity of samples were measured before and after the thermal treatment using Proceq Pundit Lab + pulse velocity instrument that uses a set of 54 kHz P-wave transducers. The core faces were extracted ensuring flat and end-parallel faces for better transducer attachment. Before each measurement, the core end-faces were wiped with dry cloth for removing dust and couplant gel was applied before attaching the transducers for a more precise measurement by reducing air pockets between transducers and core faces. ${\text{P}}$-wave velocity (${\text{P}}_{{\text{v}}}$) were averaged over 10 pulse measurements for each of the 5 samples, both before and after heating for better reproducibility.

### 3D-X-ray imaging

The 3D X-ray imaging studies were performed for visualizing mineral, pore and fracture networks in three dimensions. Individual 2D XY grayscale slices of each thermally treated blocks of size 1 × 1 × 1 cm^3^were imaged using X-Ray beam and a flat-panel detector in Zeiss Xradia Versa 520. The acquisition time for each of the slice was set to be 15 s at 110 kV working voltage after a careful evaluation of the image contrast and detector counts. The voxel size was kept at 14.2 μm for the imaging to resolve the thermal cracks and mineral components of the granites at highest possible resolution while imaging the entire specimen. The grayscale images are formed based on the attenuation properties of the individual components in granite. While the pores and fractures appear dark in color, the minerals with dense electron clouds appear as bright pixels. Successive 2D images were stacked together to form a 3D volume using Thermo Fisher Scientific Avizo 8.1^61^, which were later used for further analysis.

### LPGA studies

Low-pressure adsorption technique was used for determining mesopore surface area, pore volume, pore size distribution (PSD) and fractal characteristics. Prior to the adsorption experiments, powdered samples were first degassed at 110 °C, at near-vacuum (10^–4^ torr) for 12 h to get rid of trapped moisture and other volatiles. 99.9995% pure N_2_ gas was used for the adsorption study and was conducted using Quantachrome Autosorb iQ2 physisorption analyzer at a condensation temperature of N_2_ (77 K) at 1 atm.

## Results and discussion

### Mineral composition of granite

**Fig. 2:**
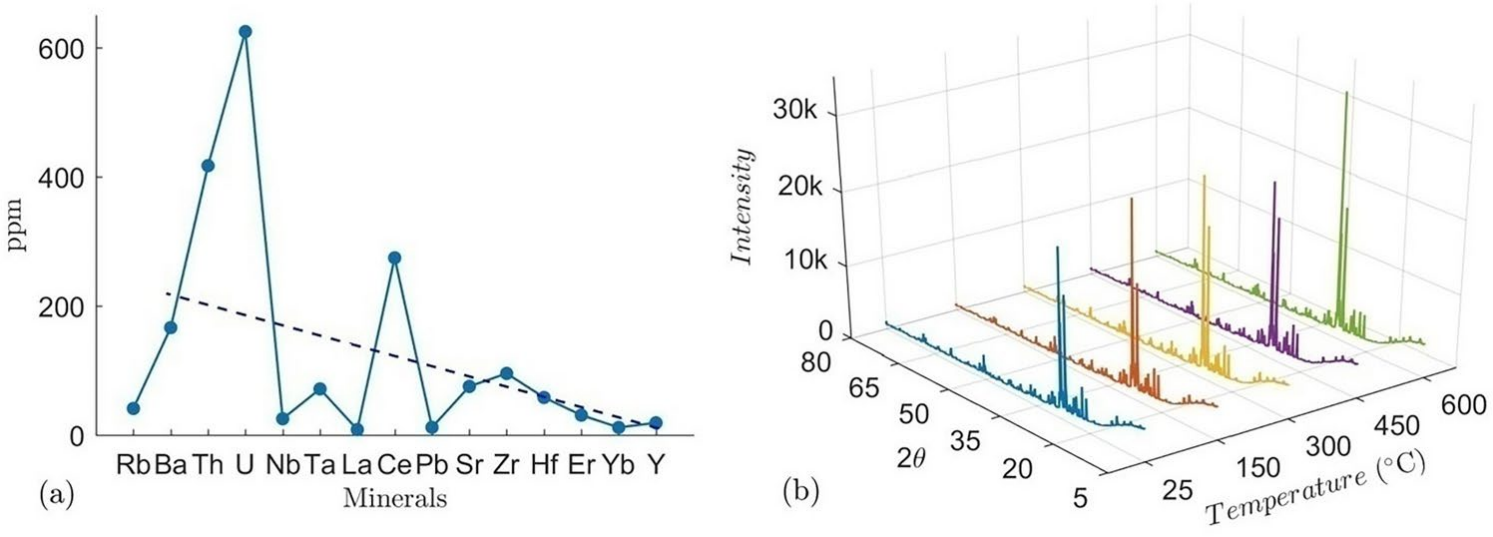
(**a**) Trace element composition determined from XRF and The dashed line represents the overall declining trend in the concentration of minerals across the sample. (**b**) X-ray diffraction profiles of the granite samples at different temperatures, illustrating the phase composition and peak intensity variations with thermal treatment.

The mineral composition, as well as major oxide and trace element concentrations, were determined using a combination of XRD and XRF respectively. The XRD plots of 5 different batches of granites at different temperatures show very sharp peaks of α-quartz, orthoclase and plagioclase feldspars and biotite, with traces of clinochlore (Fig. 2). The major oxides determined by XRF are SiO_2_, Al_2_O_3_, Fe_2_O_3_, Na_2_O, K_2_O, CaO, FeO, MgO and MnO (Table 2). Prior to thermal treatment, the unheated rock samples were carefully examined through thin-section petrography and whole-rock XRF/XRD analyses to establish their initial petrological and mineralogical context. Macroscopically, the rock displays a medium- to coarse-grained, holocrystalline texture consistent with a typical granitic lithology.

**Table 2:** Major oxide composition and respective concentrations of the granite as determined by XRF along with mineralogical composition with volume abundance.

Composition	Concentration (ppm)	Composition	Concentration (ppm)	Mineral	Vol %
SiO_2_	683,300	Na_2_O	77,800	Quartz	1.78
TiO_2_	8800	K_2_O	18,900	Plagioclase	71.01
Al_2_O_3_	186,300	P_2_O_5_	3000	Orthoclase	10.73
Fe_2_O_3_	27,200	Sr	547	Diopside	12.96
FeO	44,800	Ba	401	Hypersthene	0.21
MnO	900	Ni	7	Ilmenite	0.87
MgO	17,800	Cr	28	Magnetite	1.87
CaO	67,500	Zr	369	Apatite	0.53

Major Minerals: Plagioclase constitutes the dominant feldspar phase (> 70 vol%) and typically occurs as subhedral to euhedral grains with minimal visible alteration. Orthoclase/K-feldspar (~ 10 vol%) is present as larger subhedral crystals, frequently intergrown with quartz or showing perthitic textures. Quartz (~ 2 vol%) appears largely interstitial, forming anhedral grains that fill the interstices between feldspar crystals. Minor to trace amounts of diopside, hypersthene, magnetite, ilmenite, and apatite are dispersed throughout the matrix, commonly concentrated near grain boundaries. Small amounts of carbonate minerals and pyrite occur in thin veins or localized pockets, suggesting later-stage hydrothermal alteration or fluid flow rather than primary magmatic crystallization. These carbonate veins and pyrite inclusions do not constitute a major fraction of the rock; however, they do indicate some degree of secondary overprint that may influence porosity or crack connectivity under thermal stress. Plagioclase crystals typically exhibit well-defined cleavage planes, whereas orthoclase often appears as partially altered, subhedral crystals with minor fractures. Quartz grains maintain a mostly clean, unstrained appearance, except for rare microcracks near mineral boundaries. The accessory minerals are small and randomly distributed, with no continuous network of alteration seams evident in the untreated sample. Collectively, these features reflect a predominantly felsic magmatic protolith with limited hydrothermal overprint. This initial characterization provides the baseline against which thermally induced transformations are later assessed.

In thin section, the plagioclase crystals often appear subhedral and display well-defined cleavage planes, while orthoclase typically occurs as larger subhedral grains. Quartz grains, although present at lower volume fractions, commonly fill interstitial spaces among feldspar crystals, exhibiting anhedral morphologies. Mafic minerals (e.g., diopside and hypersthene) are dispersed as discrete grains, reflecting a localized enrichment in ferromagnesian components. Accessory phases such as magnetite and ilmenite appear sporadically and are typically associated with the feldspar or quartz boundaries. Minor carbonate and pyrite are occasionally observed in thin veins or pockets, suggesting a degree of post-emplacement hydrothermal alteration. However, the overall mineral framework remains largely intact, indicating that these secondary mineralizations are localized and do not extensively modify the primary magmatic fabric.

The rock samples are enriched with radioactive minerals and rare earth elements. Figure 2a shows that the rock is enriched in Zr, U, Th, and Nb (at low concentrations). The high level of rare earth elements (REEs) in upper mantle indicates that the mantle is heterogeneous and has a depleted/enriched discontinuity at a lower depth. The elements Yttrium (Y) and Ytterbium (Yb) are commonly used to indicate the depth. The“negative anomaly”on the graph at the Y-Yb section, indicates a deep source (Fig. 2a). Conversely, it also refers to a relative depletion in the concentration of elements (in this case Y and Yb) compared to neighboring rare earth elements. The high abundance of feldspar and quartz in Chumathang granite contributes to its thermal stability, making it a suitable candidate for EGS. Additionally, the enrichment of rare earth elements and radioactive minerals may influence geochemical interactions during geothermal operations, potentially affecting fluid-rock interactions and long-term reservoir performance. Such geochemical variations can modify surface reactivity, potentially altering mineral dissolution rates and precipitation processes, thereby impacting fluid flow paths and heat transfer efficiency in the reservoir over the long term.

### P-Wave velocity and thermal damage factor

**Fig. 3:**
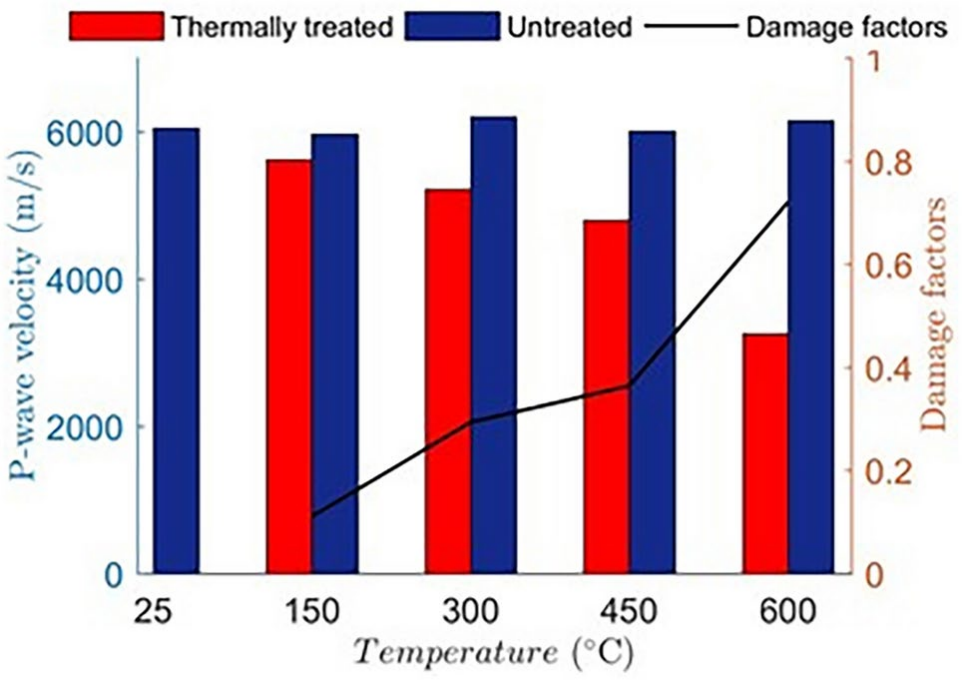
P-wave velocity of Chumathang granite samples before and after the thermal treatment and the trend of damage factor with increasing temperature (black line).

Figure 3 shows the P-wave velocities and the thermal damage factor in the samples at different temperatures. It can be noticed form the figure that the initial P-wave velocity of all untreated specimens was around 6000 m/s. After temperature treatment, thermal cracks are generated in the samples, which gradually increases with increasing temperature. The thermal cracks development indicates that the rock is damaged by the heating, leading to a slow-down of the travel speed of the P-waves. The thermal damage is calculated after considering that the strain–stress relationship is destroyed by thermal treatment. The modified relationship for stress–strain can be written as:

1 $$\sigma = E_{T} \varepsilon$$

where,$E_{T} = E(1 - D_{f} )$, is the elastic modulus of materials damaged by thermal treatment. The thermal damage factor $D_{f}$ can be written as:

2 $$D_{f} = 1 - \frac{{E_{T} }}{E}$$

The elastic modulus of rock can be written as:

3 $$E = \frac{{\rho V_{p}^{2} (1 + \nu )(1 - 2\nu )}}{1 - \nu }$$

where $\rho$, $\nu$ and $V_{p}$ are the density, Poisson’s ratio and P-wave velocity, respectively. Substituting Eq. 3 in to Eq. 2, we have

4 $$D_{f} = 1 - \left( {\frac{{Vp_{T} }}{{V_{p} }}} \right)^{2} \frac{{\rho_{T} (1 + \nu_{T} )(1 - 2\nu_{T} )(1 - \nu )}}{{\rho (1 - \nu_{T} )(1 + \nu )(1 - 2\nu )}}$$

Given that measured variations in lateral and longitudinal deformations and density were within the experimental error margins, these factors have been neglected for the simplified calculation of the damage factor. The Eq. (4) is further reduced to

5 $$D_{f} = 1 - \left( {\frac{{V_{pT} }}{{V_{p} }}} \right)^{2}$$

where $V_{p}$ and $V_{pT}$ are the P-wave velocities in untreatedand thermally treated samples, respectively. From the Fig. 3, it was found that the thermal damage factor increases with an increase in temperature, and thus, structural damage of the samples increases with temperature by formation of thermal cracks. The significant reduction in P-wave velocity observed between 450 $^\circ{\rm C}$ and 600 $^\circ{\rm C}$ indicates the progressive formation and propagation of thermal microcracks, which compromise the elastic integrity of the granite matrix. This increase in the thermal damage factor aligns with existing studies, reinforcing the correlation between elevated temperatures and seismic property degradation in granitic reservoirs. When corrections for lateral and longitudinal deformations and density variations are applied, the magnitude of the damage factor adjusts marginally; however, the overall trend of increasing damage with temperature is maintained.

### 3D X-ray CT microscopy

**Fig. 4:**
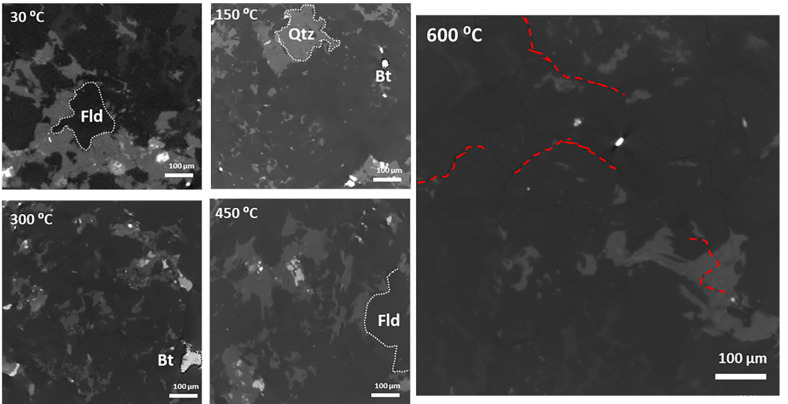
XY slices of X-ray CT images of thermally treated granite samples. The dominant compositions i.e. quartz (Qtz), feldspars (Fld) and biotite (Bt) has been shown in the images. Thermal cracks are distinctly visible at 600 °C and are marked with red dotted lines.

High resolution X-ray CT imaging was a crucial part of this study in visualizing the mineral framework in the granites and also resolving the thermal cracks and their disposition in the granite samples treated at high temperatures. Minerals were easy to identify in individual 2D slices of the grayscale CT images based on their brightness, which can be further correlated with their respective density. Density-dependent X-ray intensity is represented as:

6 $$\frac{I}{{I_{0} }} = e^{{ - \left( {\frac{\mu }{{\rho_{m} }}} \right)\lambda }}$$

which is a modified Beer-Lambert law^61^, where ${I \mathord{\left/ {\vphantom {I {I_{0} }}} \right. \kern-0pt} {I_{0} }}$ is the X-ray intensity per unit length of material, ${\mu \mathord{\left/ {\vphantom {\mu {\rho_{m} }}} \right. \kern-0pt} {\rho_{m} }}$ is the mass attenuation coefficient which is a ratio of attenuation coefficient $\left( \mu \right)$ and mass density $\left( {\rho_{m} } \right)$. $\lambda$ is the inelastic mean free path of the X-ray, which depends on the thickness of the material, perpendicular to the source of X-ray. After minor contrast and exposure adjustments, mineral components of the granites are easily visible (Fig. 4), with the thermal cracks prominently visible in 600 °C heated samples.

Acquired image slices were merged using a filtered back-projection algorithm, which is a discretized version of inverse Radon transform. X-ray CT imaging can induce several artefacts and noises, which may create bias while image analysis. Hence, post-processing of the 3D volumes was performed to reduce common artefacts generated due to beam hardening and sensor noises formed from low detector pixels^62,63^. A low-pass median filter was applied on the 3D volume to normalize pixel intensity based on a neighboring algorithm. Overuse of median filter may cause loss of intensity information of the pixels, posing difficulty in segmentation. Based on the image contrast and the presence of noise, we limited use of median filter to three iterations. We enhanced the image details by using a technique called linear high-pass unsharp masking. In this process, a blurred and inverted copy of the image is overlaid on the original, which makes the fine details more noticeable. Optimized application of unsharp masking drastically improved the edge contrast, and in this case helped in a better delineation of different minerals and cracks. Intricate thermal crack networks are predominantly present within feldspar-rich domains, highlighting the role of mineralogical heterogeneity in fracture propagation mechanisms. These detailed microstructural observations are critical for understanding permeability evolution and optimizing stimulation strategies in EGS applications.

#### Feature isolation

**Fig. 5:**
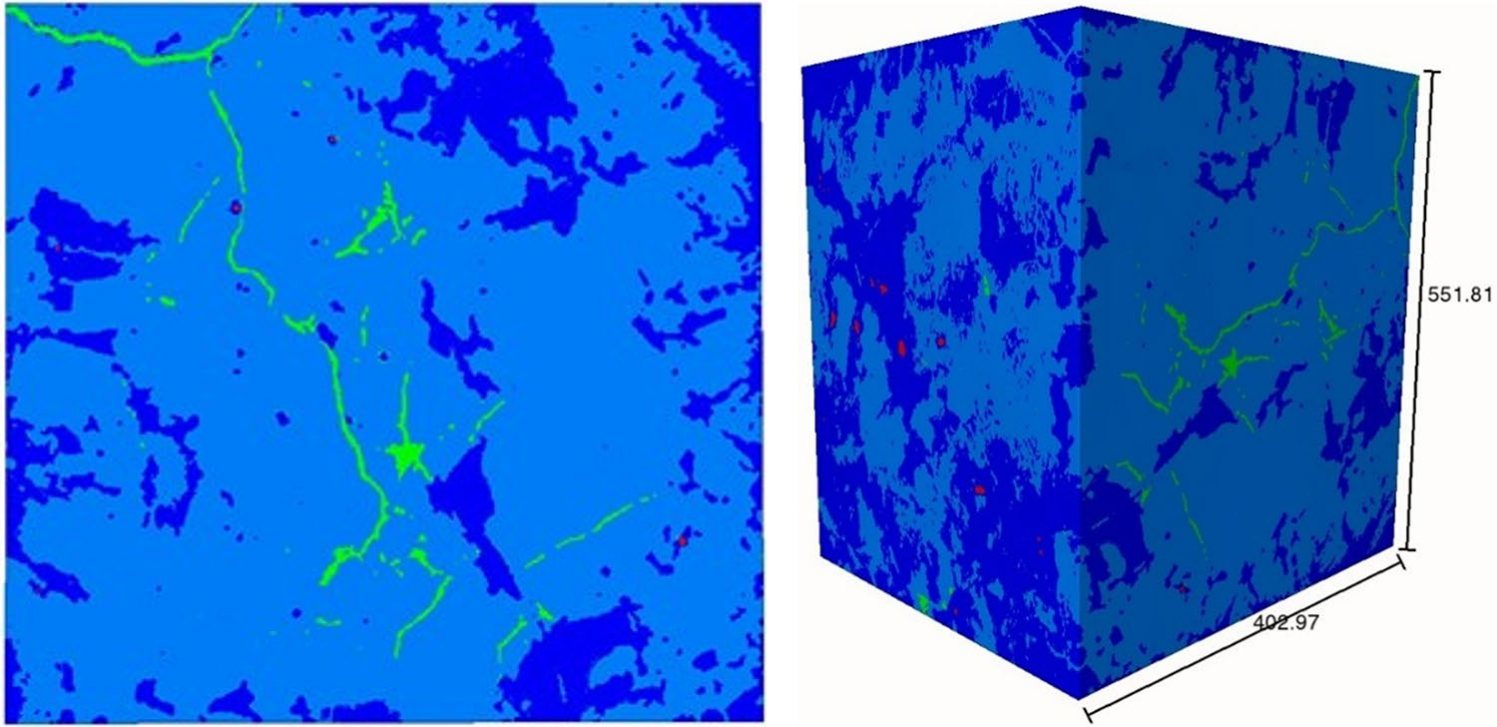
Thresholded and labelled 2D image slice (left) and 3D volume showing fractures (green), feldspars (light blue), quartz (deep blue) and biotite (red) for the 600 °C heat-treated sample.

Mineral compositions as well as thermal cracks at high temperature were visible in the reconstructed X-ray CT volumes (Fig. 4). For a proper quantification, it was essential to isolate each of the features from the reconstructed 3D volume. All post processing and image based quantification were performed with Avizo 8.1^64^. The segmentation method opted for this study included thresholding of the images to isolate features of separate intensity. Biotite shows the highest intensity due to its high density, followed by quartz and feldspar; fractures and cracks are essentially void spaces, which show the lowest intensity than others (Fig. 4). Labelled data were generated using global thresholding for each 3D volumes, as imaging condition and exposures differ slightly from one sample to other, hence selecting uniform thresholding parameters for all samples may lead to errors and on the other hand, setting threshold for each 2D slices is time consuming. We have segregated mainly four key features from the images, i.e. quartz, feldspars, biotite and fractures/cracks with the last feature visible distinctly in the 600 °C heated sample. After assigning threshold values to label fields, the watershed was applied to smoothen the threshold margin between different features, facilitating better segmentation without any loss of information (Fig. 5).

**Fig. 6:**
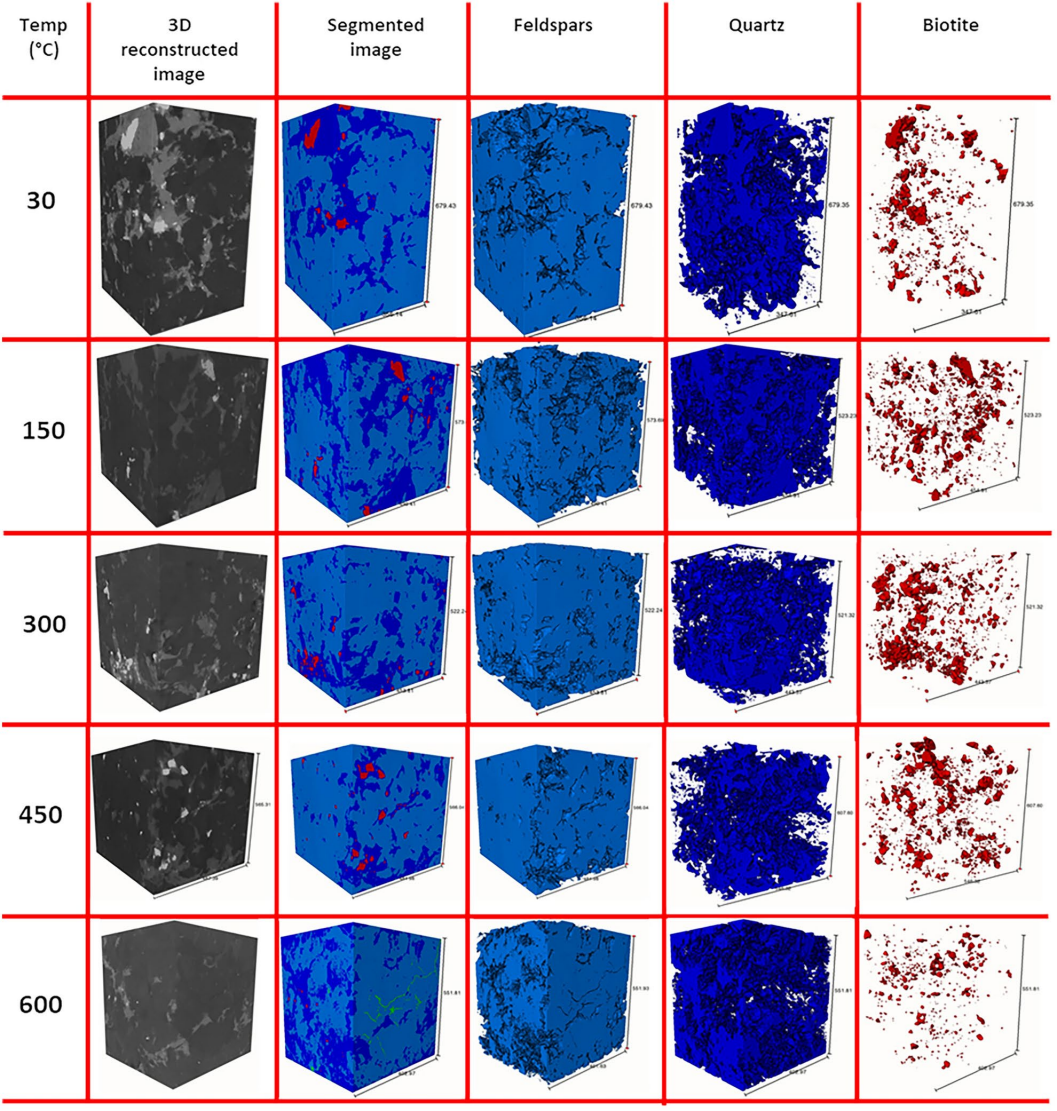
Extracted 3D volume fraction of individual minerals from the thermally treated granites.

Biotite shows the highest intensity, followed by quartz and feldspar; fractures and cracks are essentially void spaces, which show the lowest intensity than others (Fig. 4). Isolated fractions of individual minerals (Fig. 6) suggest the relative abundance of quartz, feldspar and biotite, with feldspar being the most dominant and biotite being the least. In most of the cases, biotite inter growths are found in quartz rather than in feldspars. Since quartz is a more resistive mineral than feldspar, biotite enclosed in quartz are naturally protected by the host during future hydrothermal or magmatic events.

#### Feature quantification

**Fig. 7:**
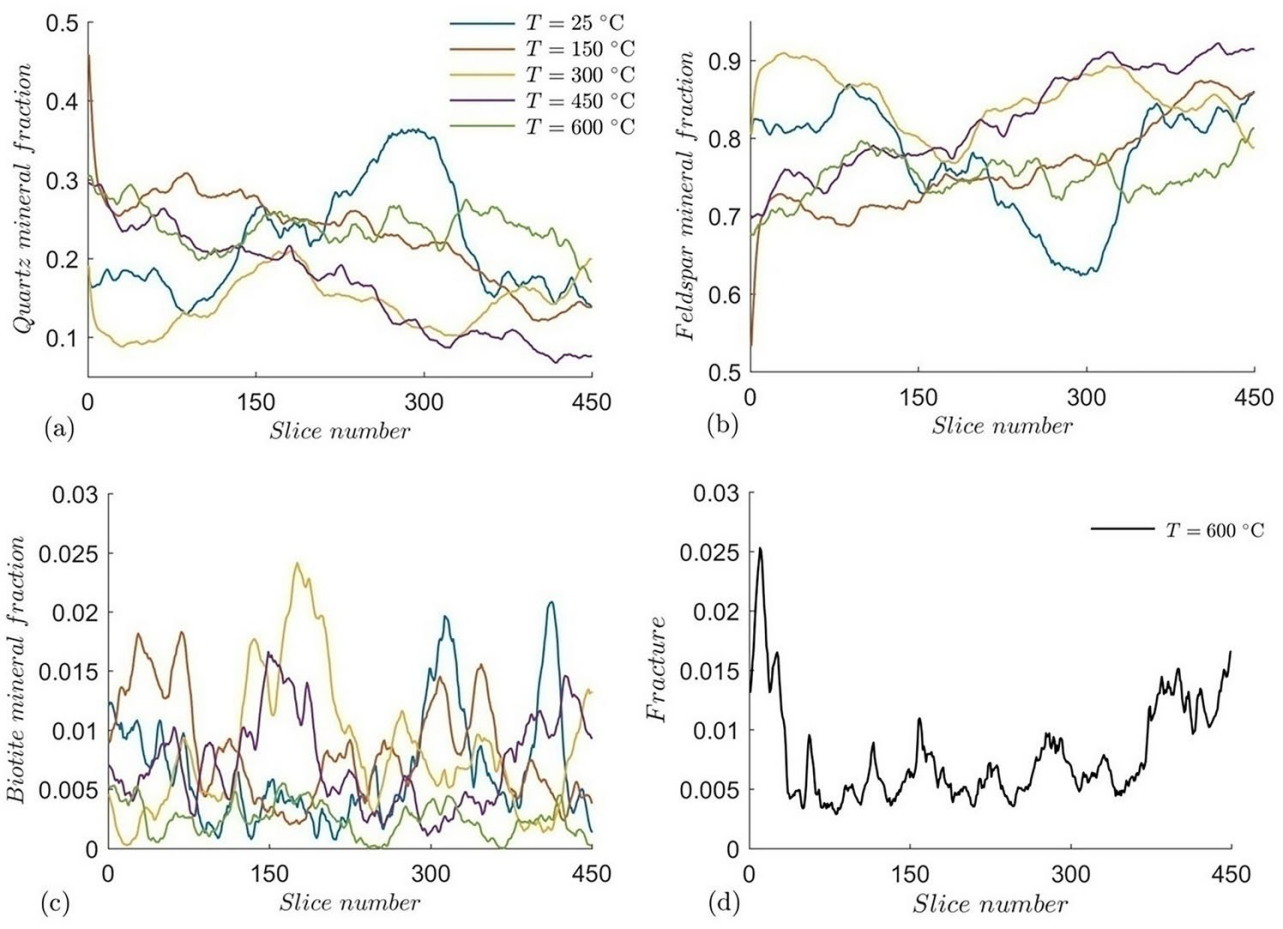
XY-slice wise volume fraction of individual mineral components and fractures of the granites at different temperature.

The relative abundance of individual mineral components and fractures in each slice is shown in Fig. 7. The biotite fragments are haphazardly distributed, but show close resemblance to the quartz abundance in each slice. Similarly, the fracture abundance resembles the feldspar volume fraction, supporting the fact that fractures are concentrated in feldspar domain.

**Fig. 8:**
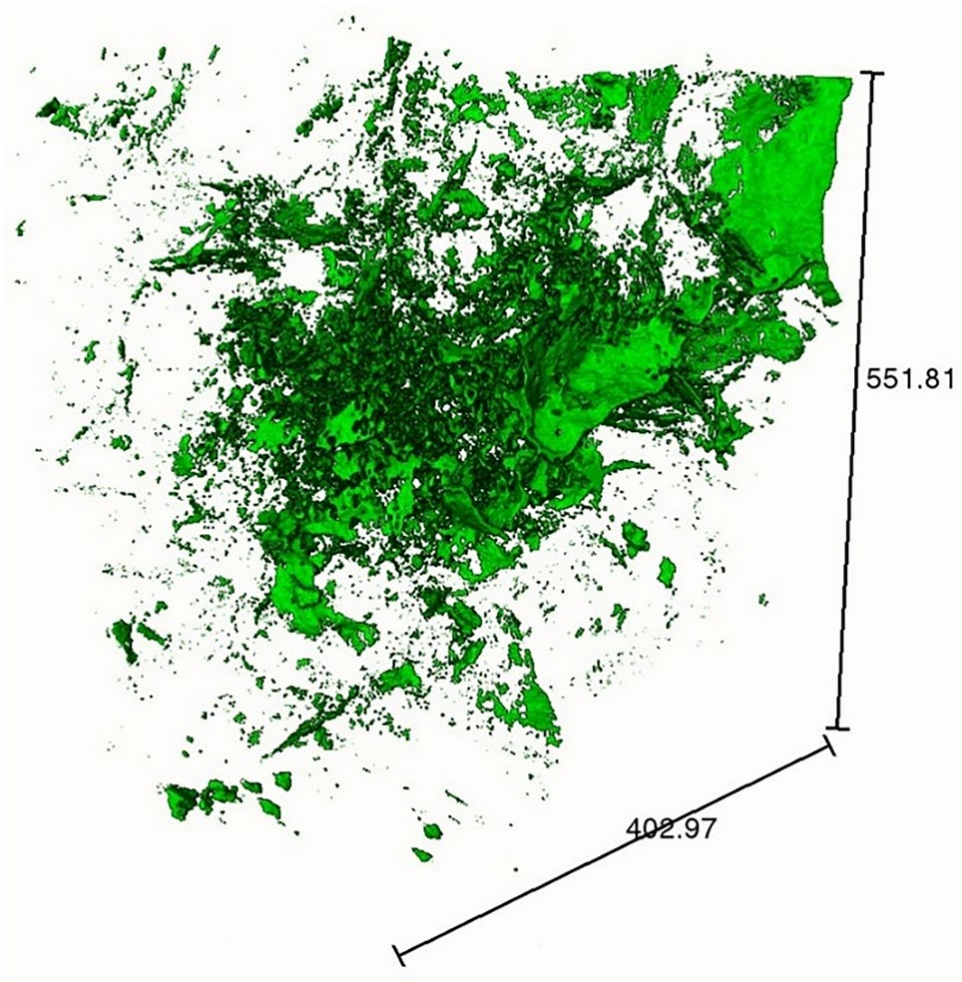
Fracture network extracted from the 600 °C heat-treated sample showing haphazard orientations (scale units in microns).

The thermal cracks are clearly visible in the samples heated at 600 °C, but are not visible in samples at lower temperatures. Previously, granites were reported to produce thermal cracks at or above 600 °C^37^ upon slow-cooling and at or above 300 °C upon rapid cooling. The interesting fact of the thermal cracks is their localization, majorly in the feldspar-rich regions. In some cases, these cracks have truncated into quartz grains (Fig. 4), but the biotite grains have rarely been affected by the thermal cracks (Fig. 5). The thermal expansion of these minerals plays a major role in guiding the fracture propagation. Quartz and micas have around four times higher thermal expansion rate than feldspars (an average of 37, 35.4 and 10.4 for quartz, mica and feldspars respectively, all units in 10^–6^ K^−1^)^65^. Moreover, phase boundaries between quartz-feldspar and quartz-mica can cause microcracks at a higher temperature^66^. The flaky arrangement of mica causes expansion along the c-axis, however, random orientation of weak planes in quartz causes a structural anisotropy during thermal strain resulting in a disorganized fracture network in the feldspar fraction, as it accommodates the strain imposed by quartz and biotite (Fig. 8). A two-component system tested by Vollbrecht et al.^67^, involving feldspar mantle and surrounding a quartz core resulted in brittle fractures in the feldspar fraction. Quantitative assessment revealed that thermal cracks are primarily localized within feldspar-rich regions, suggesting that mineral composition significantly dictates fracture network architecture. This selective fracturing behavior has important implications for reservoir permeability and the efficiency of heat extraction processes in EGS.

### Low pressure gas adsorption

**Fig. 9:**
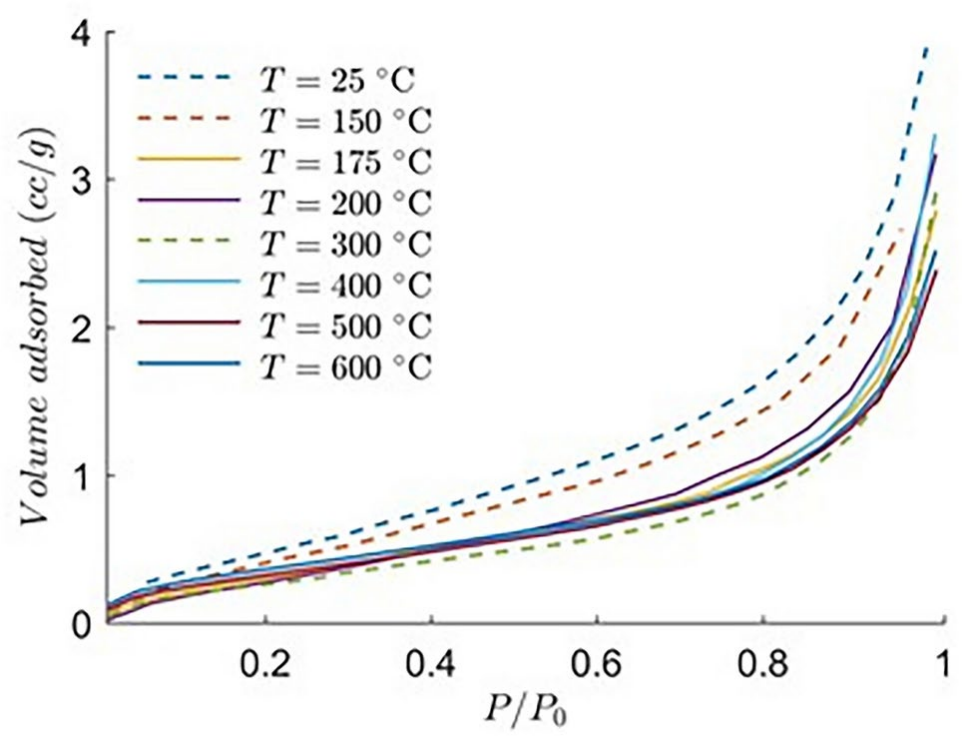
N_2_ adsorption isotherms of the thermally treated granites.

The N_2_-LPGA studies were performed to characterize the granite mesopores. N_2_ molecules, with its quadrupolar moment can attach itself to active sites of mesopores due to Van der Waals forces between them. Based on the energy potential of active sites, multiple layers of N_2 _molecules can attach themselves to each site, creating successive films of condensed gas until the pores are saturated^68^. In a low-pressure gas adsorption study, adsorbate gas is dosed from a very low relative pressure to saturation pressure, which is 1 atm in this case. Relative pressure is represented as P/P_0_, where P is the user assigned equilibrium pressure at each point and P_0_ is the condensation pressure. For our study, we selected 20 points between a relative pressure range of 0.01 to 0.99 to construct the adsorption isotherms (Fig. 9). All our pore attributes were calculated on the basis of the adsorption isotherms, the desorption branch was avoided. From Fig. 9, it is observed that N_2_ adsorption capacity decreases with increase in temperature, from room temperature to 300 °C in this case. However, with further increase in temperature from 300 to 600 °C, a minor increase of nitrogen adsorption capacity was noted, indicating a possible increase in concentration of macropores or formation of microfractures in the granite samples. It can be further observed for all temperature ranges, substantial amount of nitrogen adsorptions occurred at high relative pressure, quite evident from the slope of the adsorption isotherm. This is a typical behavior of a mesoporous media and suggests that at higher relative pressure, the granite samples possess a significant amount of mesoporosity (Fig. 9).

#### Mesopore attributes

**Fig. 10:**
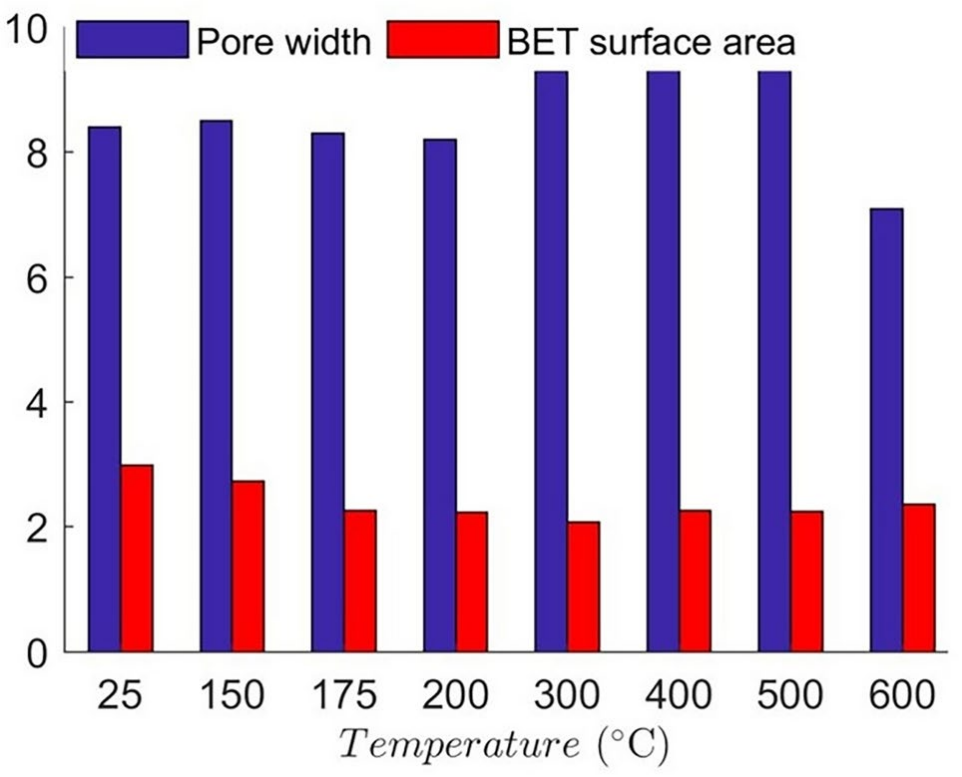
BET surface area (m^2^/g) and pore width (nm) of the granite samples at different temperatures.

Surface area of mesopores was calculated using the multi-point Brunauer–Emmett–Teller (BET) method^69,70^ taking the adsorption points between a relative pressure range of 0.03 to 0.5. The BET surface model can be represented as

7 $$\frac{1}{{\left[ {V_{a} \left( {\frac{{P_{0} }}{P} - 1} \right)} \right]}} = \frac{{P\left( {C - 1} \right)}}{{P_{0} V_{m} C}} + \frac{1}{{V_{m} C}}$$

where ${P \mathord{\left/ {\vphantom {P {P_{0} }}} \right. \kern-0pt} {P_{0} }}$ is the relative pressure as mentioned before, $V_{a}$ is the volume of gas adsorbed at standard temperature and pressure, $V_{m}$ is the volume of gas adsorbed to form a monolayer and $C$ is a constant related to the enthalpy of adsorption. The BET surface area of granite at all temperatures are presented in Fig. 10, which indicates that the BET surface area of granite decreases faster with increasing temperature ranging from room temperature to 175 °C, which is conformable with the adsorption isotherms. The slight increase of BET surface area of granite in heating from 300 to 600 °C is likely related to thermally induced mesopore concentration. Higher temperature may form thermal cracks and may connect several mesopores to form a bigger macropore, simultaneously creating new mesopores, resulting in the increasing surface area past 300 °C. An average pore width follows an inverse relation with the surface area with the 400 °C heated samples showing the highest pore width (9.5 nm) and 600 °C heated granite showing the lowest (7.1 nm). This conforms to the X-ray CT observations, where new thermally induced fractures increase the macropore fraction by joining multiple larger mesopores, while micropores combine to form smaller mesopores, thereby reducing the average pore width.

#### Pore size distribution

**Fig. 11:**
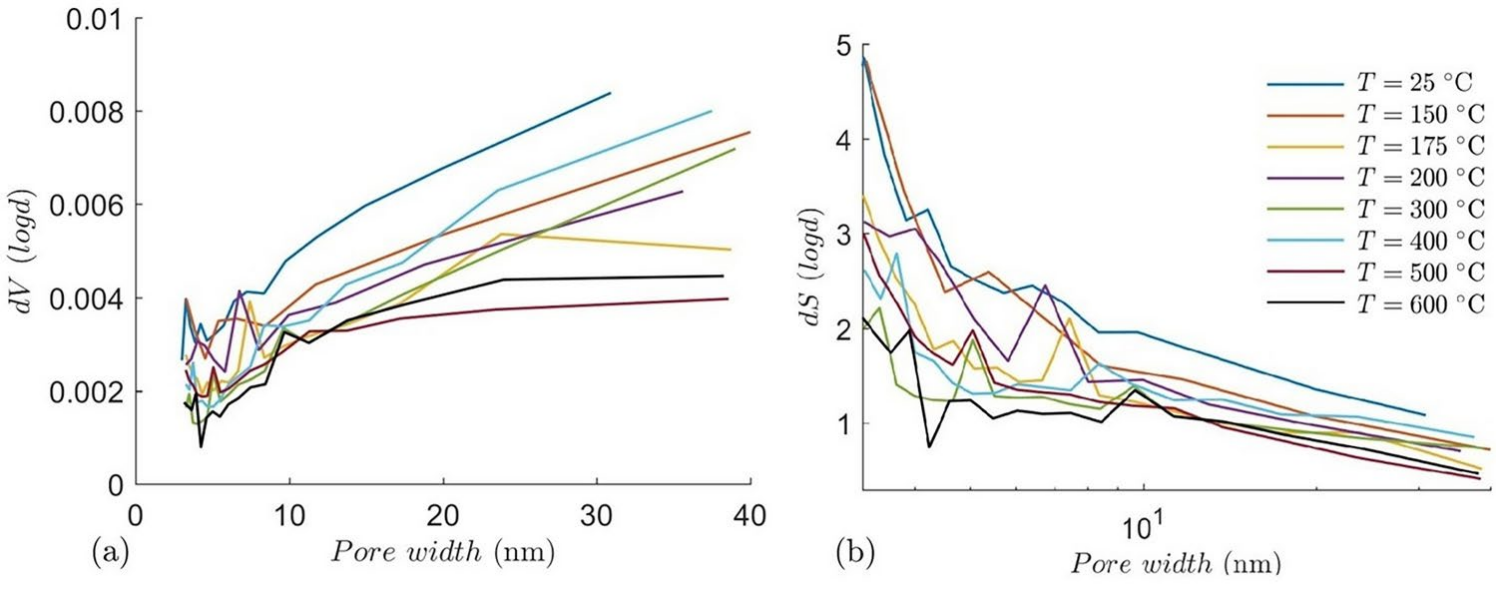
(**a**) BJH pore size distribution and (**b**) differential surface area of the granite samples after thermal treatment.

Pore size distribution of the thermally treated granites were determined from the adsorption branch of the N_2_ isotherm using Barrett-Joyner-Halenda (BJH) method. N_2_ upon silica model was chosen for calculating the PSD which shows that larger mesopores are more abundant compared to smaller mesopores, with cumulative pore volume decreasing with increasing temperature (Fig. 11). The room temperature sample has the highest pore volume, whereas the granite pretreated at 500 $^\circ{\rm C}$ shows least pore volume, mainly in the coarser mesopore range. It is to be noted that the pore size range of the LPGA method is limited up to 200 nm, hence larger pores and cracks are beyond its limit. However the drop in mesopore and smaller macropore volume with temperature indicates more larger crack formation and coalescence of smaller pores, which is also supported by the elastic wave measurement. This shift in pore size distribution may enhance fluid circulation and heat transfer efficiency within the granite matrix. This trend is consistent with the observed formation of thermal cracks that bridge existing pores, thereby modifying the overall pore architecture to support improved geothermal performance. PSDs show an increasing pore volume with increasing pore width, with few kinks at the smaller mesopore range. The BJH differential surface area shows that smaller mesopores possess higher surface area, whereas larger pores contribute less (Fig. 11).

#### Fractal dimension

**Fig. 12:**
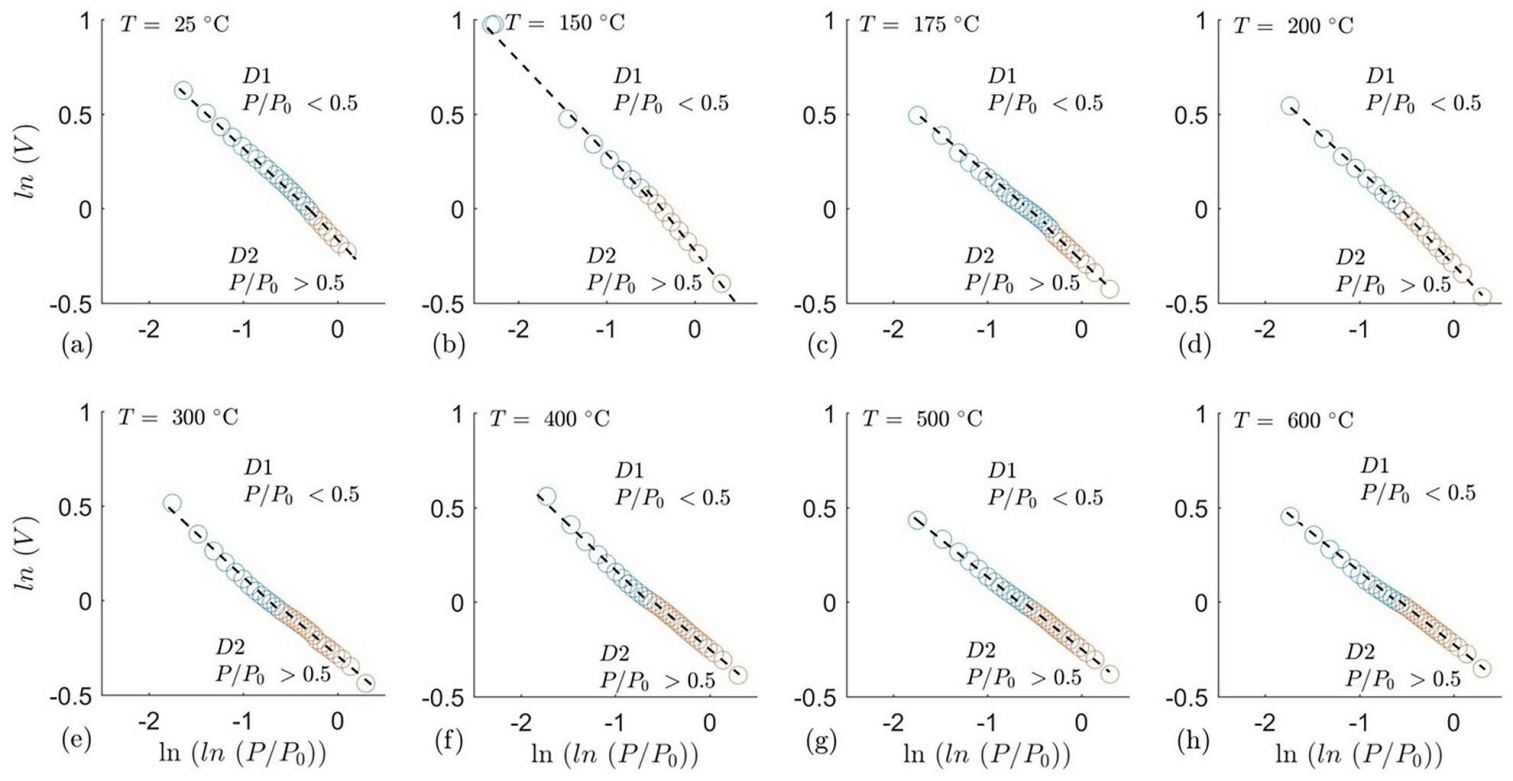
FHH fractal dimension of the thermally treated granites. Orange datapoints indicate finer mesopores, whereas blue datapoints indicate coarser mesopores. The dotted lines represent the linear fitting trend.

The fractal geometry of granite mesopores helps to characterize their porous structure and surface irregularities, and to determine the influence of those structural features on the gas adsorption behaviors. The fractal dimension concept was first introduced by Mandelbrot^71^to characterize the surface roughness of a porous particle. The fractal dimension is greater than the topological dimension but must be less than equal to the dimension of the embedding Euclidean space^72^. As the surface roughness increases, fractal dimension varies from 2 to 3, where 2 indicates a perfectly smooth surface, and 3 represents a very rough surface. Several methods exist to calculate the fractal dimension of geological material using low-pressure nitrogen adsorption data, of which, the Frenkel-Halsey-Hill (FHH) model is the most widely applied. The modified equation of FHH for multilayer adsorptive model can be written as (Pfeifer and Avnir^73^)

8 $$\ln \left( {\frac{V}{{V_{m} }}} \right) = A\,\ln \left[ {\ln \left( {\frac{{P_{0} }}{P}} \right)} \right] + {\text{constant}}$$

where $V$,$V_{0}$,$A$,$P$ and $P_{0}$ are the volume of nitrogen adsorbed gas molecules, the volume of monolayer coverage, the power law exponent which depends on the fractal dimension $(D)$ and the mechanisms of adsorption, the equilibrium pressure and the saturation pressure of the gas, respectively. The slope of the line A/S was calculated following the above equation (Fig. 12).

The adsorption mechanism is dependent on capillary condensation in the coarser mesopore region, whereas in finer mesopores, Van der Waals force is more dominant. Fractal dimension in the finer mesopores ($D_{1}$) can therefore be calculated (Table 3) from the slope ($S_{1}$) of the straight line in the $\ln V$ versus $\ln \left( {\ln \left( {{{P_{0} } \mathord{\left/ {\vphantom {{P_{0} } P}} \right. \kern-0pt} P}} \right)} \right)$ FHH plot using equation:

9 $$D_{1} = 3 + S_{1}$$

**Table 3:** Fractal dimensions calculated from the slope of the fitting trends for the thermally treated granites.

Temperature ($^\circ{\rm C}$)	$R_{1}$	$S_{1}$	$D_{1}$	$R_{2}$	$S_{2}$	$D_{2}$
25	0.99	−0.52	2.48	0.99	−0.25	2.25
150	0.99	−0.57	2.43	0.99	−0.25	2.25
175	0.99	−0.49	2.51	0.99	−0.22	2.34
200	0.99	−0.54	2.46	0.99	−0.21	2.37
300	0.99	−0.42	2.58	0.99	−0.19	2.43
400	0.99	−0.42	2.58	0.99	−0.18	2.46
500	0.99	−0.41	2.59	0.99	−0.18	2.46
600	0.99	−0.39	2.61	0.99	−0.18	2.46

In the coarser mesopores, where capillary condensation is more dominant, the equation can be modified as:

10 $$D_{2} = 3 + S_{2}$$

where $D_{2}$ and $S_{2}$ are fractal dimension and slope for coarser mesopores respectively.

It can be clearly seen in Table 2 that $D_{1}$, which represents smaller mesopores (2-8 nm width) generally is a higher value than $D_{2}$, which represents larger mesopores (8-50 nm width). This signifies that smaller mesopores have rougher pore walls as compared to larger mesopores, there by translating to an increased surface area in smaller mesopores (Fig. 11). It is also interesting that $D_{1}$ and $D_{2}$ both show minor increase with an increase in temperature, thereby indicating increase in roughness in the pore walls. The plateauing of $D_{2}$ suggests that the pore surface complexity reaches a saturation point beyond 400 °C, indicating that additional thermal treatment does not further enhance surface roughness, which has implications for fluid flow dynamics and heat transfer efficiency in geothermal reservoirs.

Our results show that changes in pore geometry, connectivity, and the development of fracture networks under thermal stress provide key insights into the transport and mechanical properties of the rock. These observations offer valuable parameters that can be incorporated into numerical reservoir models, aiding in the optimization of fluid circulation, heat transfer, and mechanical stability within EGS heat exchangers. Although our study was conducted under laboratory conditions, the quantified parameters such as the evolution of mesopore structures and the propagation of thermal cracks are critical for upscaling these findings to field-scale applications. This enhanced understanding facilitates more accurate predictions of reservoir performance and supports the design of more efficient and robust EGS operations.

## Conclusions

This multi-proxy study of high-enthalpy granites collected from Chumathang geothermal field provides an insight into the pore attributes, thermal response of the granites, and their interrelations. Thermal treatment of granite up to 600 °C, followed by slow cooling can result in the creation of microfractures at intermediate temperatures and prominent and more pervasive thermal cracks at a higher temperature, leading to a rapid decrease in the P-wave velocity. The thermal cracks are more localized in feldspar-rich domains with occasional penetration in quartz grains while biotite remains unaffected. This phenomenon results due to varying thermal expansion of these minerals and the accommodation of thermal strain by feldspars. Consequently, mesopore abundance decreases with increasing temperature as pores often get connected to form larger macropores/cracks with increasing temperature. Although, a general increase in surface area and fractal dimensions can be observed in samples with increasing temperatures. Interestingly, smaller mesopores have rougher surfaces and higher fractal dimensions thereby contributing most to the total surface area. Although the pore size distribution of the granites represents a lesser fraction of smaller mesopores in total porosity, a general assumption can be made that with a fixed porosity, smaller pores will be more effective in terms of higher surface area generation compared to coarser pores.

## Data Availability

All data generated or analyzed during this study are included within this published article.
